# *Hypericum perforatum* L. and the Underlying Molecular Mechanisms for Its Choleretic, Cholagogue, and Regenerative Properties †

**DOI:** 10.3390/ph16060887

**Published:** 2023-06-15

**Authors:** Ala Mohagheghzadeh, Parmis Badr, Abdolali Mohagheghzadeh, Shiva Hemmati

**Affiliations:** 1 Department of Pharmaceutical Biotechnology, School of Pharmacy, Shiraz University of Medical Sciences, Shiraz 71468-64685, Iran; alamhz1376@gmail.com; 2 Pharmaceutical Sciences Research Center, Shiraz University of Medical Sciences, Shiraz 71468-64685, Iran; badrparmis@gmail.com (P.B.); mohaghegh@sums.ac.ir (A.M.); 3 Department of Phytopharmaceuticals (Traditional Pharmacy), School of Pharmacy, Shiraz University of Medical Sciences, Shiraz 71468-64685, Iran; 4 Biotechnology Research Center, Shiraz University of Medical Sciences, Shiraz 71468-64685, Iran; 5 Department of Pharmaceutical Biology, Faculty of Pharmaceutical Sciences, UCSI University, Cheras, Kuala Lumpur 56000, Malaysia

**Keywords:** bile acid, bioactive compounds, cholesterol, *p*-coumaric acid, hypericin, hyperforin, medicinal plant, microbiota, natural products, ursodeoxycholic acid

## Abstract

Any defects in bile formation, secretion, or flow may give rise to cholestasis, liver fibrosis, cirrhosis, and hepatocellular carcinoma. As the pathogenesis of hepatic disorders is multifactorial, targeting parallel pathways potentially increases the outcome of therapy. *Hypericum perforatum* has been famed for its anti-depressive effects. However, according to traditional Persian medicine, it helps with jaundice and acts as a choleretic medication. Here, we will discuss the underlying molecular mechanisms of *Hypericum* for its use in hepatobiliary disorders. Differentially expressed genes retrieved from microarray data analysis upon treatment with safe doses of *Hypericum* extract and intersection with the genes involved in cholestasis are identified. Target genes are located mainly at the endomembrane system with integrin-binding ability. Activation of α5β1 integrins, as osmo-sensors in the liver, activates a non-receptor tyrosine kinase, c-SRC, which leads to the insertion of bile acid transporters into the canalicular membrane to trigger choleresis. *Hypericum* upregulates CDK6 that controls cell proliferation, compensating for the bile acid damage to hepatocytes. It induces ICAM1 to stimulate liver regeneration and regulates nischarin, a hepatoprotective receptor. The extract targets the expression of conserved oligomeric Golgi (COG) and facilitates the movement of bile acids toward the canalicular membrane via Golgi-derived vesicles. In addition, *Hypericum* induces SCP2, an intracellular cholesterol transporter, to maintain cholesterol homeostasis. We have also provided a comprehensive view of the target genes affected by *Hypericum*’s main metabolites, such as hypericin, hyperforin, quercitrin, isoquercitrin, quercetin, kaempferol, rutin, and *p*-coumaric acid to enlighten a new scope in the management of chronic liver disorders. Altogether, standard trials using *Hypericum* as a neo-adjuvant or second-line therapy in ursodeoxycholic-acid-non-responder patients define the future trajectories of cholestasis treatment with this product.

## 1. Introduction

Bile acids are biological surfactants synthesized from cholesterol in the liver. These steroidal molecules are involved in various cell-signaling pathways [1]. The bile acid-activated nuclear receptors, including farnesoid X receptor (FXR), pregnane X receptor (PXR), constitutive androstane receptor (CAR), and vitamin D receptor (VDR), have a vital role in the regulation of bile secretion and energy expenditure [2]. Bile is formed at the hepatocytes and travels through the bile canaliculi into the gallbladder [3]. Any defects in bile formation, secretion, or flow may lead to the accumulation of highly cytotoxic molecules in the hepatocytes and give rise to cholestasis. Chronic cholestasis will eventually bring about liver fibrosis, cirrhosis, and subsequent hepatocellular carcinoma (HCC) [4]. Besides idiopathic etiologies, drugs, ischemia, stenosis, pregnancy, viruses, bacteria, and fungi, the two typical cholangiopathies are primary biliary cholangitis (PBC) and primary sclerosing cholangitis (PSC). While both intra- and extrahepatic large bile ducts are affected by PSC, any damage to the intra-hepatic small bile duct leads to PBC [5,6].

Various treatments have been adopted in order to inhibit the progression of cholestasis. Alteration in the bile flow rate, bile synthesis, and bile detoxification are reported adaption mechanisms to resolve cholestasis. One of these treatments is to use an FXR ligand to reduce inflammation and hepatic bile acid production. There are fibroblast growth factor 19 mimetics that regulate bile acid synthesis [7]. Through competitive removal of toxic hydrophobic bile acid molecules, ursodeoxycholic acid (UDCA) prevents damage to hepatocytes and bile ducts. UDCA, with a dose of 13–15 mg/Kg/day, is a multifunctional medication in PBC management [8]. However, more than 40% of the patients do not respond to UDCA and are considered to experience severe hepatic complications within 10 years [9,10]. Hence, a precise and helpful outcome of UDCA, as the first-line treatment, in the various stages of PBC, is considered doubtful, and the efficacy of second-line treatments has been investigated in trials [11,12,13,14]. UDCA seems not to be promising in unfavorable consequences of intrahepatic cholestasis of pregnancy [15]. It also elevates the morbidity and mortality rate in cholestatic neonates and infants [16] and might be intolerable in some patients due to its side effects, such as diarrhea, nausea, and vomiting [17]. Required evidence on the helpful impact of UDCA for other hepatic afflictions such as PSC or metabolic liver disorders is insufficient [10]. While UDCA is not beneficial in PSC, obeticholic acid (OCA) is the only approved alternative for PBC patients who are UDCA-intolerant or non-responders [18]. To attenuate the extrahepatic manifestation of cholestasis, such as pruritis, cholestyramine, rifampicin, naltrexone, and sertraline, might be prescribed. Fibrates and statins are recommended to regulate dyslipidemia [19]. Antioxidants might be also proposed to protect the liver from the damage of bile acids [20,21,22]. Diverse immunomodulators have been tested for cholestatic liver disorders [9]. Glucocorticoid medications such as prednisolone and budesonide as anti-inflammatory agents, and fibrates that, besides triggering biliary secretion, show anti-inflammatory properties and mycophenolate mofetil are some of the immunomodulators that might be used as alternatives to UDCA [23]. However, long-term uses of immunosuppressants are not recommended due to side effects [23]. The utmost treatment is liver transplantation, restrained by the shortage of organ donor candidates with the hazard of post-transplantation complications [6]. These observations fueled the ongoing controversy on the complete efficacy of UDCA in hepatobiliary disorders, and adjuvants or second-line therapies are profoundly necessary.

Plants are known as a rich source of pharmaceutically valuable primary and secondary metabolites [24,25]. *Hypericum perforatum* L., as one of the most famous medicinal herbs in the world, is reported to ameliorate liver-related disorders, including hepatic steatosis, impaired hepatic lipid metabolism, and cholestasis [26]. This plant is known as St. John’s wort (SJW) (*Hypericum* or Millepertuis), and is a member of the Hypericaceae (Clusiacaeae) family. It is a herbaceous perennial plant indigenous to Europe, Western Asia, and Northern Africa [27,28,29]. Its flowers are yellow to coppery, having four or five petals, many stamens, and a pistil. Bioactive compounds are stored as a red-colored liquid in golden petals [28]. Pedanios Dioskourides (a famous herbalist) was the first to introduce *Hypericum* as a medicinal plant in the first century. *Hypericum* flowers and aerial parts, including its leaf, are the main parts that are consumed [30,31]. Today, dried aerial parts of *Hypericum* are commercially available as tablets, capsules, tea, and tinctures for their therapeutic potential [28,31,32]. Low cost, good accessibility, high efficacy, low major side effects, high tolerability, and a lack of dependence in comparison with conventional anti-depressants are commonly reported reasons for self-treatment with *Hypericum* [33].

One of the main therapeutic approaches in treating cholestasis is increasing the bile flow (choleretic effect) and excreting excess bile by feces (cholagogue effect). *Hypericum* has been suggested for ileus or intestinal obstruction, and also biliary obstruction in Traditional Persian Medicine (TPM) [34]. According to TPM, *Hypericum* is a natural bile laxative with choleretic and cholagogue effects. Both seeds and leaves are cholagogue agents and increase bile secretion [34]. In addition, according to Turkish folk medicine, *Hypericum* can help with jaundice, hepatic, and biliary disorders [35]. Within this study, we aimed to describe the most important pharmacological effects and specialized metabolites of *H. perforatum*. Then, we will discuss the target genes associated with *Hypericum*’s total extract and bioactive compounds involved in its cholagogue, choleretic, and regenerative properties. We believe that a comprehensive view of the underlying molecular mechanisms of *Hypericum* is promising in targeting multiple pathways and lights a new scope in the management of liver chronic disorders such as cholestasis, fibrosis, and HCC.

## 2. Results and Discussion

### 2.1. Hypericum Medical Attributes and Its Bioactive Compounds

*Hypericum* has been used traditionally for the treatment of several disorders such as mental illness, insomnia, gastrointestinal tract diseases, skin wounds, eczema and burns. It is now one of the most greatly studied medicinal herbs in the world [36]. For example, in TPM, its vaginal formulation, as an emmenagogue, increases menstrual flow [34]. The urethral dosage form has a diuretic effect [37]. The alcoholic extract of the leaves relieves sciatica pain and gout [38]. Topical formulations have antimicrobial effects, useful for wound healing, particularly burn wounds. *Hypericum*’s aerial parts are widely used to treat mild to moderate depression, anxiety, irritability, neuralgia, sciatica, viral infections including HSV, vaginal problems such as vaginal atrophy and vaginal dryness, menopausal complications, and cystitis [30,39]. Many investigations have approved that *Hypericum* has anti-inflammatory [40], anti-tumor, anti-bacterial (Gram-positive bacteria), anti-oxidative, and neuroprotective effects in Parkinson’s and Alzheimer’s disease [41]. The SARS-CoV-2 virus resulted in a global pandemic called COVID-19, and various naturally derived compounds could target this pathogen [42,43]. This virus can be affected by the antiviral properties of *Hypericum* and its metabolites [44]. *Hypericum* is also utilized widely as a natural source of food flavor [45]. A growing interest has been observed in enriched or functional foods in the last few decades [46]. There is increased attention to *Hypericum*, as a mood enhancer, to be added to different foods, including cookies [47], functional beverages [48], and probiotic beverages [49]. Its antioxidant activity for long-term storage of food products has been also reported [50]. The most dominant pharmacological properties of *Hypericum* are summarized in Table 1.

**Figure 1:**
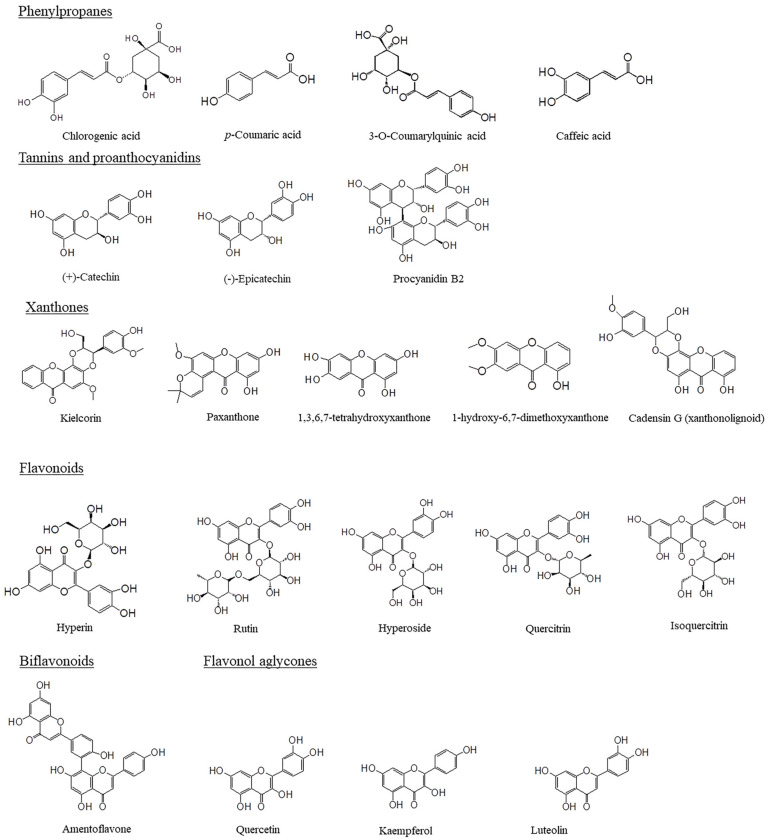

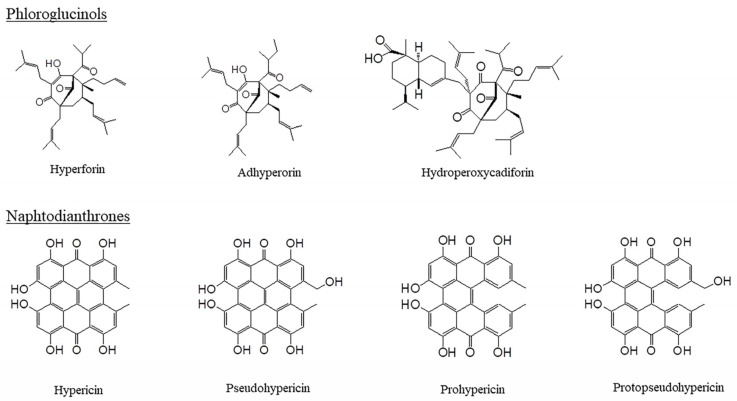
Chemical structures of the main specialized metabolites of *Hypericum perforatum*.

Concerning its diverse medical attributes, *Hypericum* is abundant in bioactive metabolites such as flavonoids, phenylpropanes, xanthones, tannins, proanthocyanidins, phloroglucinols, naphthodianthrones, biflavonoids, flavonol aglycones, and several volatile oils. The potential medical benefits of *Hypericum* are mostly attributed to naphthodianthrones and phloroglucinols (Figure 1). The naphthodianthrones in *H. perforatum* are mainly hypericin, pseudohypericin, protohypericin, pseudoprotohypericin, cyclopseudohypericin, and emodinanthrone. This plant contains several phloroglucinols, including hyperforin, adhyperforin, and hydroperoxycadiforin [30,51]. Cell, tissue, and organ culturing is an important tool for the identification and isolation of secondary metabolites [52,53]. This strategy has been employed for the propagation of *Hypericum* as an industrially vital plant for the production of hypericin [54].

**Table 1:** Various pharmacological effects of *Hypericum perforatum* L., its mode of actions, and corresponding metabolites for each effect.

Effect	Mechanism	Metabolite	Model of Study
Antidepression	NT * metabolism ↓, Regulation of NT receptors sensitivity, NT synaptic reuptake ↓	Total extract [29]	*In vivo* animal model [29]
Antioxidative	Free radical scavenger	Total extract [55,56], Rutin, quercetin [57]	*In vivo* animal model [55,56] *In vitro* assay and ex vivo rat membrane model [57]
Anti-inflammatory	NF-κb ↓, GSH ↑, Ca influx ↓, IL-6 ↓, IL-12 ↓, TNF-α ↓	Total extract [56], Pseudohypericin, Quercetin, Amentoflavone, Chlorogenic acid [58], Hyperforin [59]	*In vivo* animal model [56] RAW 264.7 macrophage cell line [58] A549 cells & *in vivo* animal model [59]
Antitumor	Apoptosis ↑	Hyperforin, Hypericin [60]	*In vivo* animal model [60]
Hypolipidemic Cholesterol ↓ Triglycerides ↓ HDL ↑	FAS ↓, ACC ↓, PPAR-α ↑	Total extract [26,61]	*In vivo* animal model [26] 3T3L1 adipocyte [61]
	HMG-CoA reductase ↓, Cholesterol excretion via secreted bile acids ↑	Total extract [62,63,64] Rutin [65]	*In vivo* animal model [62,63,64,65]
	(LP1) ↓, (Dgat1) ↓	Total extract, Rutin [61]	3T3L1 adipocyte [61]
	(Apo-A1) ↑	Total extract [66]	*In vivo* animal model [66]
Hepatoprotective (hepatic steatosis ↓) ALT ↓, AST ↓	PPAR-γ ↑	Chlorogenic acid [67]	3T3L1 preadipocyte [67]
	AMPK ↑	Hypericin [61,68], Rutin [69]	L02 and HepG2 cells & *in vivo* animal models [61,68], HepG2 cells [69]
	Free radical scavenger, Neutrophil activation	Quercetin [70], Hyperoside [71]	*In vivo* animal model [70,71]

* ACC: Acetyl-CoA carboxylase; ALT: Alanine transaminase; Apo-A1: apolipoprotein-A1; AMPK: AMP-activated protein kinase; AST: Aspartate aminotransferase; Dgat1: Diacylglycerol *O*-acyltransferase 1; FAS: Fatty acid synthase; GSH: Glutathione; HDL: High-density lipoprotein; HMG-CoA reductase: 3-hydroxy-3-methylglutaryl coenzyme-A reductase; IL: Interleukin; LP1: Lipid transfer protein 1; NT: Neurotransmitter; NF-κb: Nuclear factor kappa B; PPAR: Peroxisome proliferator-activated receptors; TNF-α: Tumor necrosis factor α. Upward (↑) and downward arrows (↓) represent increase and decrease, respectively.

#### 2.1.1. Mechanisms of Antidepressant Activity of *H. perforatum*

As the only approved herbal substitute for synthetic anti-depressants, *Hypericum* is now registered in many countries to treat mild to moderate depression with a daily dose of 500 mg, which is equivalent to 1–2 mg of hypericin [72]. Although neurotransmitter (NT) reuptake inhibitory effect and non-selective monoamine oxidase (MAO) inhibition seem to be the main pharmacological actions of *Hypericum*, the exact mode of its antidepressant activity is not well understood [73]. *Hypericum* modulates some genes, such as the corticotropin-releasing factor (CRF) involved in the hypothalamic–pituitary–adrenal axis, as the hyperactivity of this system is a sign of depression [73]. The anti-depressive effect of *Hypericum* is majorly attributed to hyperforin, hypericin, and its flavonoids [74]. Selective antagonization of CRF is observed by pseudohypericin [75]. Adhyperforin binds to serotonin and noradrenalin receptors resulting in the inhibition of NT reuptake [76]. Some *in vitro* investigations admit the MAO inhibitory effect of hypericin [77]. *In vitro* administration of hyperforin inhibits the uptake of several NTs at presynaptic terminals, including dopamine, noradrenaline, GABA, L-glutamate, and especially serotonin [78]. This observed effect is mainly due to the disruption of intracellular Na^+^ concentrations as a result of transient receptor protein potential channel (TRPPC6) activation by hyperforin [79]. Hyperforin is also believed to impair the monoamine storage at vesicles and probably affects the solute carrier (SLC) gene family, including SLC 17, 18, and 32 [78]. Taken together, considering its few side effects, *Hypericum* can be a good “household alternative” to medications such as selective serotonin reuptake inhibitors (SSRIs) or tricyclic antidepressants (TCA) [30].

#### 2.1.2. Hepatoprotective and Anti-Atherogenic Activity of *Hypericum*

Compared to a single chemical medication such as UDCA, *Hypericum* extract contains plentiful metabolites, interfering with various metabolic pathways to ameliorate cholestasis consequences. For example, in UDCA non-responsive PBC patients, biochemical indices are not improved [80]. However, according to *in vivo* animal models, *Hypericum* extract helps with hepatic biochemical markers such as ALT and AST [70]. Although UDCA reduces total cholesterol, it does not have a significant effect on triglyceride (TG), high-density lipoprotein (HDL), and low-density lipoprotein (LDL) [8]. *Hypericum* helps with hepatotoxicity, hepatitis, hepatocyte peroxidation, non-alcoholic fatty liver disease (NAFLD), hepatic lipid metabolism, and hepatic steatosis [26,68]. *Hypericum* extract lowers total cholesterol, raises HDL, and decreases TG content in *in vivo* animal models [81,82] (Table 1). Similarly, *in vivo* studies show that hyperforin can attenuate serum TG, cholesterol, and LDL levels [62]. Hyperoside, a pivotal flavonoid found in *Hypericum* species, regulates cholesterol metabolism and therefore attenuates NAFLD [83]. Since free radicals can damage cellular macromolecules, excessive production of free radicals in the liver may contribute to a variety of hepatic disorders [84]. *Hypericum* is best known for its anti-inflammatory and anti-oxidative activity, which gives a sense of hepatoprotection. *Hypericum*’s hepatoprotective activity can be attributed to the suppression of TNF-α and IL-6 cytokine contents in the liver [26,61]. These two pro-inflammatory cytokines are produced mainly by adipocytes, and their concentration adjusts the percentage and distribution of fat [85]. Similar to fibrates, *Hypericum* extract has been shown to increase the expression of PPAR-α in animal models promoting bile acid excretion [26]. There is a close connection between lysophosphatidylcholine (LysoPC) content as a hepatic inflammation biomarker and TGF-β/SMAD3 pro-inflammatory signaling pathway, which is associated with bile acid accumulation in the liver [86]. *Hypericum* is also said to regulate LysoPC content according to *in vivo* animal trials [26].

By modulating the peroxisome proliferator-activated receptor (PPAR-γ), *Hypericum* regulates the secretion and expression of adiponectin, which balances glucose and fatty acid metabolism [87]. *Hypericum* also improves glucose and lipid metabolism by inhibiting protein tyrosine phosphatase 1B catalytic activity in obese mice models [88]. The dysbiosis in gut bacteria is observed in cholestatic liver disorders due to the damage to bile-acid-sensitive species. Fecal microbiota transplantation and the use of probiotics are recommended as alternative treatments to UDCA for cholestasis [9]. It has been defined in animal models that *H. perforatum* modulates the gut microbiome and consequently normalizes serum lipid profile and reverses the hepatic steatosis symptoms [62]. Finally, to conclude a clear positioning statement, standard trials using *Hypericum* alone and in combination with UDCA as the neo-adjuvant or second-line therapy define the future trajectories of cholestasis treatment with this plant.

### 2.2. Analysis of Differentially Expressed Genes (DEGs) upon Treatment of HepG2 Cells with H. perforatum Crude Extract

**Figure 2:**
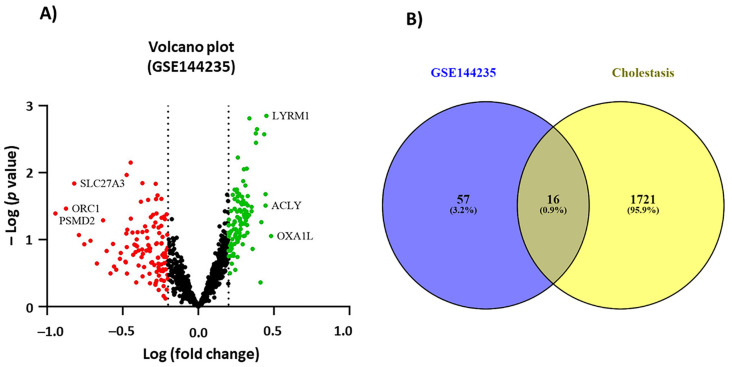
(**A**) Volcano plots related to differentially expressed genes (DEGs) of GSE144235. Differences in gene expressions of HepG2 cells treated with 2.5 µg/mL *Hypericum* extract compared to the DMSO-treated control. Each dot in the plot is a symbol of gene expression. A |log F_c_ (fold change) | > 0.2 (*x*-axis) and a *p*-value < 0.05 (*y*-axis) were set as statistical significant criteria. The red dots represent down-regulated genes and the green dots represent up-regulated genes. (**B**) A Venn diagram representing 73 DEGs upon treatment with *Hypericum* crude extract and 1737 genes related to cholestasis. The overlapped section is the symbol of mutually expressed genes in both conditions. There are 16 expressed genes related to cholestasis among 73 monitored DEGs.

A study by Ozturk et al. suggested that *Hypericum*, as a choleretic and cholagogue agent, exerts its hepatoprotective effects by increasing bile secretion and flow [35]. In addition, according to TPM, *Hypericum* has been used because of its choleretic and cholagogue activities. Bile-duct-ligated rodent models have been used as classic cholestatic *in vivo* models, which are replaced by genetically modified mice, and inducible models using toxicants [89]. Cell lines, such as human hepatoma HuH-7, known for high expression and localization of bile acid transporters and bile salt export pumps, are also appropriate models to investigate the underlying choleretic mechanisms [90]. However, due to feasible access and culture conditions, HepG2 cells are favorable models to study drugs that target hepatic functions [91]. To define the underlying mechanism of choleretic and cholagogue activities, DEGs in HepG2 cells treated with a safe amount of *H. perforatum* extract (2.5 µg/mL) or DMSO were analyzed (according to the available microarray data from VanderMolen et al. in the GEO database as described in the Section 3). Analysis of the box plot quartiles and their median reconfirms that the data distribution is valid and there is no outlier sample. A total of 73 DEGs were identified in GSE144235 considering a *p*-value < 0.05 and |log Fc (fold change)| > 0.2 as the cut-off criteria. As a result, 52 and 21 genes were up- and down-regulated, respectively, represented by volcano plots (Figure 2A) (Table S1).

### 2.3. Identification of Target Genes

A total of 1737 genes related to cholestasis were retrieved using the GeneCards database (Table S2). To define the potential choleretic and anti-cholestatic mechanisms of *Hypericum*, the intersection of 73 DEGs upon treatment with the safe dose of plant extract and 1737 genes related to cholestasis was determined and displayed using a Venn diagram (Figure 2B). A total of 16 common genes, including EZH2, SCP2, ILK, CDK6, EGF, SPP1, PLOD3, SKIV2L, CXCL2, NISCH, ICAM1, SRC, CAT, COG7, BAX, and COG4 were identified.

For additional investigation on the function and mechanism, all the sixteen retrieved genes were imported to DAIVID as a functional enrichment analysis tool. Gene Ontology (GO) enrichment results are listed in Table 2. The table includes MF, BP, and CC, which refer to molecular function, biological process, and cellular components, respectively, ranking by *p*-value. GO enrichment analysis revealed that the most significant biological pathways for enriched genes were involved in the regulation of MAP kinase activity, as well as lipid and metabolic processes. It seems that *Hypericum* regulates P38 mitogen-activated protein kinase (MAPK). The MAPK signaling pathway regulates various hepatic functions, including bile acid synthesis, bile acid excretion, and bile-acid-induced apoptosis, and involves in the pathogenesis of hepatic steatosis. MAPK also regulates the insertion and retrieval of the bile salt export pump (BSEP) and the multidrug-resistance-associated protein 2 (MRP2) into the plasma membrane [92]. Hence, *Hypericum* as a choleretic agent prompts the insertion of these transports by regulating MAPK. Regarding cellular components and molecular function, DEGs were mainly at the endomembrane system with integrin binding ability. The top KEGG pathway analysis of mutual genes revealed that these genes are mainly related to lipid and atherosclerosis as well as EGFR tyrosine kinase inhibitor resistance.

### 2.4. Protein–Protein Interaction (PPI) Network of DEGs

**Figure 3:**
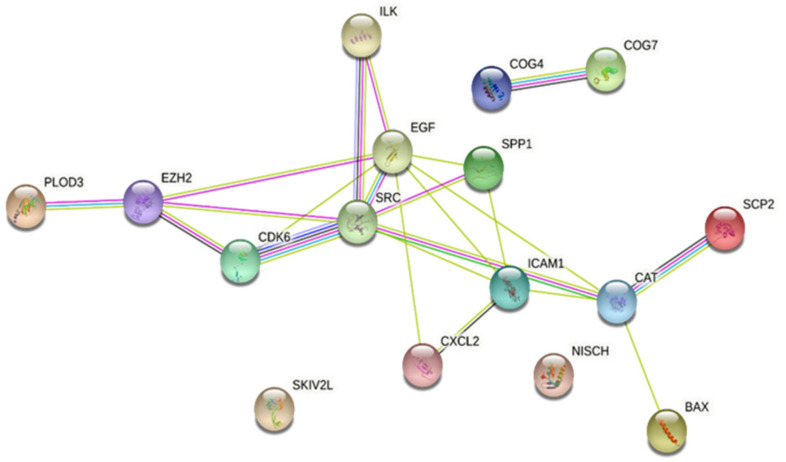

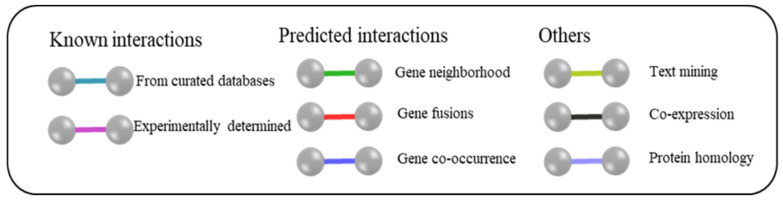
Protein–protein interaction (PPI) network of DEGs in treated groups with *Hypericum* extract in common with the genes involved in cholestasis.

**Figure 4:**
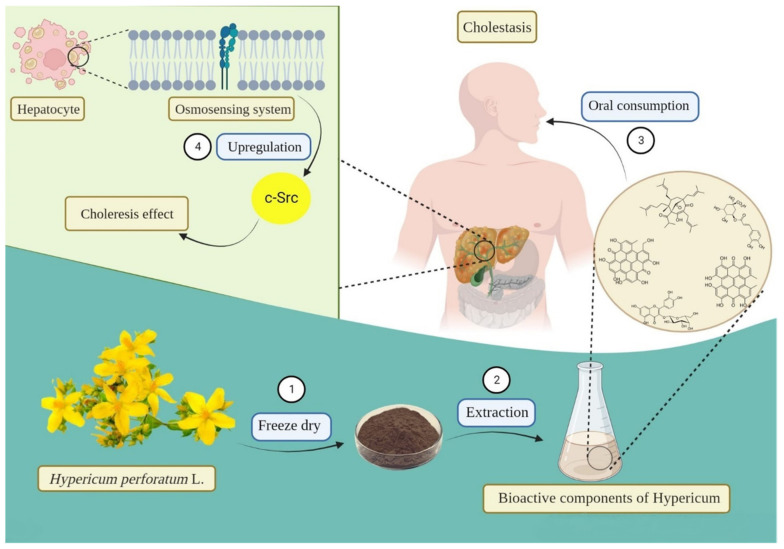
The role of c-Src in the choleretic effect of *Hypericum perforatum*. The up-regulation of c-Src by *Hypericum* extract eventually results in the insertion of the bile salt export pump and multidrug resistance protein 2 as two important bile acid transporters into the canalicular membrane and trigger choleresis.

**Figure 5:**
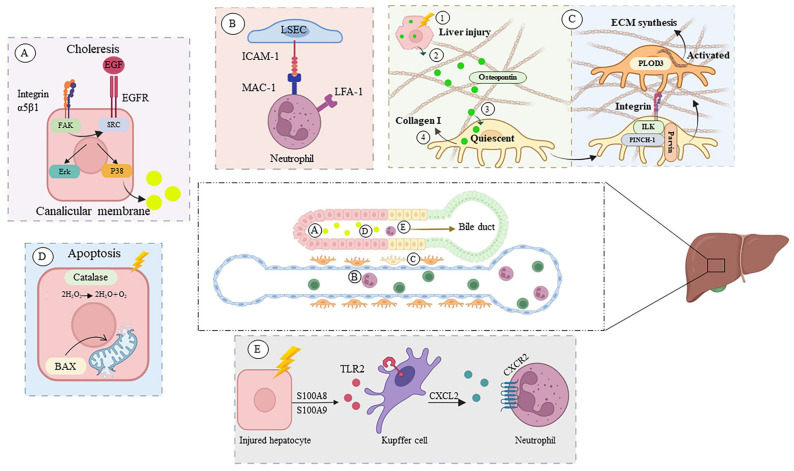
A summary of the function of cholestasis-related genes affected by *Hypericum* extract (**A**) EGFR activation by EGF or any EGFR ligands leads to activating SRC and eventually induces choleresis (**B**) ICAM-1 is an expressed binding molecule on neutrophils, which mediates the neutrophil recruitment to the inflammatory sites (**C**) OPN expression in the liver helps with liver regeneration, regulating hepatic infiltration of macrophages and neutrophils. When a ligand binds to integrin, the structure of integrin changes, and ILK binds to the tail of integrin, resulting in the activation of the ternary complex of ILK, PINCH, and PARVIN. As a result of activating this signaling pathway, HSCs become activated, motile, and synthesize more ECM components, including collagen type I, α-actin, and fibronectin. PLOD3 generates hydroxylysine-linked carbohydrates, which are important for collagen biosynthesis (**D**) Cholestasis can cause apoptosis and necrosis in hepatocytes and cholangiocytes. Following oxidative stress and increased accumulation of ROS, cholestasis induces BAX expression. BAX, as a pro-apoptotic member of the Bcl-2 family, activates caspases, and releases mitochondrial inter-membrane space (IMS). Catalase is increased in cholestasis, which is due to the increased demand for hepatocyte detoxification from excess production of H_2_O_2_ (**E**) TLR2/S100A8/S100A9 signaling pathway is introduced as hepatic CXCL2 regulator, which results in neutrophil recruitment.

A PPI network of 16 overlapped DEGs after treatment with *H. perforatum* extract and cholestasis is observed in Figure 3. The interaction was ranked by a degree method through CytoHubba. *Hypericum* can upregulate Src, a non-receptor tyrosine kinase, as an essential member of the SRC family kinases. The SRC family kinases are of significant importance thanks to regulating many liver functions, such as bile flow and bile formation [93,94]. Anisoosmotic hepatocyte volume changes regulate the expression of SRC family kinases. Hepatocyte swelling caused by ambient hypo-osmolarity will lead to the rapid activation of the integrin system as osmo-sensors in the liver. Activation of β1 and α5β1 integrins activates the upstream c-Src, epidermal growth factor receptor (EGFR), mitogen-activated protein kinase (MAPK), focal adhesion kinase (FAK), and phosphatidylinositol kinase 3 (PI3 kinase). This will expedite the downstream activation of extracellular signal-regulated kinases (Erks) and p38 mitogen-activated protein kinase (p38 MAPK). The downstream activation of Erk and p38 will lead to the insertion of the bile salt export pump (BSEP) and multidrug-resistance-associated protein 2 (MRP2) as two important bile acid transporters into the canalicular membrane and triggers choleresis (Figure 4 and Figure 5A) [94]. The other cholestasis-related genes affected by *Hypericum* are discussed further.

- Epidermal growth factor (EGF)

The EGF gene encodes a polypeptide of 6 kDa that belongs to the epidermal growth factor superfamily. *Hypericum* upregulates EGF as a mitogenic factor that binds specifically to the epidermal growth factor receptor (EGFR), which activates several signaling pathways, including JAK/STAT, Ras/ERK, and PI3K/AKT [95]. EGFR signaling is characterized as a hepatoprotective factor and prevents cholestatic liver injury [96]. Studies have shown that ablation of the EGFR signaling pathway results in aggravated bile-acid-induced liver injury, collagen deposition in periportal areas, and upregulation of the bile acid biosynthesis enzyme. The STAT3 pathway regulates the EGFR signaling pathway, IL6, and IGF-1 and protects the liver from hepatocyte apoptosis and cholestatic injury. Due to EGFR’s role in regulating compensatory cell proliferation mechanisms, EGFR ablation will result in defective liver regeneration. The EGFR signaling pathway is also used therapeutically to prevent liver injury and fibrosis [97]. Previous studies have shown that pretreatment of hepatocytes with bile acids, including deoxycholic acid (DCA), activates the EGFR signaling pathway. EGFR activation by DCA was transduced to the MAPK pathway via Ras and the PI3 kinase pathway, as expected when hepatocytes are treated with natural EGFR ligands. Consequently, bile acid’s toxic potential is limited by itself (Figure 5A) [98].

- Intercellular Adhesion Molecule-1 (ICAM-1)

As a result of cholestasis and activation of the MAPK signaling pathway, early growth response factor 1 (Egr-1) is activated, which plays a crucial role in the progression of inflammation. In cholestasis, Egr-1 is the main inducer of ICAM-1 overexpression [99]. According to many studies, ICAM-1 expression in sinusoidal and perisinusoidal areas in cholestatic patients is higher than that in healthy controls [100]. ICAM-1 encodes a cell surface glycoprotein, named intercellular adhesion molecule 1 (ICAM-1) which binds to different types of integrins. The expression of ICAM-1 on HSCs and liver sinusoidal endothelial cells (LSEC) is critical for the attachment of leukocytes to the site of injury for the alleviation of cholestatic symptoms [100]. Excess bilirubin, bile salts, and overexpression of TNF-α in the cholestatic liver will lead to the activation of Kupffer cells [101]. Following this, hepatocytes, HSCs, and endothelial cells become involved due to TNF-α release. Neutrophils are recruited to inflamed liver sites by platelet-activating factor (PAF) and monocyte chemotactic protein-1 (MCP-1) secreted by activated HSCs. HSCs will also express ICAM-1 which helps with the neutrophil adhesion to the endothelial cells. ICAM-1 participates in many adhesion pathways, including MAC-1, and LFA-1 (expressed binding molecules on neutrophiles’ surface) to mediate neutrophil recruitment into hepatic inflamed sites (Figure 5B) [102]. Several studies have evaluated the role of neutrophils as the largest population of circulating leukocytes in the pathogenesis of many liver diseases [103]. Liver sinusoids are regularly patrolled by neutrophils, with a few resident neutrophils. Neutrophil infiltration is a necessary step in confronting liver diseases; however, excess neutrophil infiltration and activation will lead to chronic inflammation and subsequent organ dysfunction. In fact, neutrophils have been introduced as a promising target for treating several liver diseases [104]. Hepatocyte mitogens, IL-6, and TNF are released by neutrophils when they bind ICAM-1, which is a liver regeneration stimulus [104].

- Integrin-linked kinase (ILK)

The extracellular matrix (ECM) plays an important role in hepatocyte differentiation, motility, and liver regeneration. The architecture of ECM also influences hepatocyte-specific gene expression. These developmental and differentiation signals transmit to the cell interior from ECM via integrins and signal-transducing molecules. There are some ECM–hepatocyte adhesion proteins, including integrin-linked kinase (ILK) and focal adhesion kinase (FAK), that facilitate the communication between ECM and cells [105]. As the name implies, ILK encodes the intracellular kinase linked with integrin. The ILK ternary complex consists of ILK, PINCH-1, and PARVIN, which transmit ECM signals via the binding of ligands (such as collagen type I) to integrins. When a ligand binds to integrin, the structure of the integrin changes and ILK binds to the tail of integrin, resulting in the activation of the ternary complex of ILK, PINCH, and PARVIN. As a result of activating this signaling pathway, hepatic stellate cells (HSCs) become activated, motile, and synthesize more ECM components, including collagen type I, α-actin, and fibronectin (Figure 5C) [106,107]. Many cellular processes, including hepatocyte differentiation, growth, migration, and survival depend on ILK expression and its interaction with the extracellular matrix [108]. Researchers have shown that knocking down ILK results in dedifferentiated hepatocytes that can be regenerated by adding hydrated complex matrix preparations (Matrigel^®^), indicating that ILK contributes to maintaining extracellular microarchitecture. It is convincing that ILK protects cells from anoikis because of its role in the ECM, and knocking it out leads to uncontrolled apoptosis [109].

- Secreted phosphoprotein 1 (SPP1)

SPP1, located on chromosome 4 in humans, encodes a highly negatively charged protein named osteopontin (OPN) [110]. Depending on the number of post-translationally modified residues, OPN has a molecular weight ranging from 41 to 75 kDa. OPN is a multi-functional protein that is associated with ECM maintenance and turnover. It is composed of three integrin-binding domains, two heparin-binding domains, and one CD44 binding domain. By binding to these domains, signaling pathways that are involved in cell proliferation, adhesion, motility, invasion, and fibrosis are triggered. In the liver, OPN plays a role as a cytokine-like protein that is involved in fibrinogenesis [111]. The highest levels of OPN expression in the liver can be found in cholangiocytes, followed by macrophages, hepatocytes, sinusoidal endothelial cells, HSCs, and natural killer cells (NKs). OPN is related to integrin and ERK signaling pathways. OPN expression in the liver helps with liver regeneration, regulating hepatic infiltration of macrophages and neutrophils, and activation of signaling pathways, including IL6 and STAT3 in Kupffer cells (Figure 5C) [111]. Hepatic inflammation is associated with OPN expression, which serves as a chemoattractant of macrophages and neutrophils. Additionally, overexpression of OPN during liver inflammation activates integrin molecules and CD44 signaling pathways for macrophage migration, dendritic cell maturation, and T cell activation and differentiation [112].

Several studies indicate that OPN expression in liver cells is associated with PPAR-α inhibition; therefore, there is a positive correlation between OPN serum levels and hepatic cholesterol and triglycerides [113]. In addition, OPN serum levels are correlated with subsequent liver fibrosis in chronic alcoholics. OPN expression increases the wound healing ability of HSCs, as well as collagen type I production and the PI3K-pAkt-NFB pathway activation. Normal biliary epithelial cells (BECs) express OPN, whereas liver injury induces OPN overexpression, suggesting a pathogenic role for OPN in cholangiopathies [114]. Based on a microarray analysis in intrahepatic cholangiocarcinoma (ICC) patients, the most expressed gene is SPP1, which has a positive correlation with tumor size [114]. According to studies, extracellular OPN is related to obesity and lipid synthesis. OPN knockdown reduces inflammation of adipose tissue and insulin resistance in high-fat diets, suggesting that OPN contributes to metabolic syndrome. OPN^−/−^ mice demonstrate increased hepatic cholesterol after injection of recombinant OPN, but decreased levels of cytochrome P450 family-7 subfamily-A member-1 (CYP7A1), indicating that OPN regulates bile acids, which play a role in hepatic lipogenesis [111].

- Procollagen-Lysine,2-Oxoglutarate 5-Dioxygenase 3 (PLOD3)

PLOD3 encodes lysyl hydroxylase 3 (LH3), which belongs to the PLOD family (PLOD1-3) [115]. Due to its galactosyl- and glucosyl-galactosyl-transferase activities, LH3, also known as PLOD3, is the only isoenzyme that can also generate hydroxylysine-linked carbohydrates, which are important for collagen biosynthesis (Figure 5C) [116,117]. As a major component of the ECM, collagen promotes hepatic microenvironment hemostasis both biochemically and physically, making PLOD3 even more important. There are many studies demonstrating that a few weeks after a hepatic injury, the activity of lysyl hydroxylase increases, which is an indicator of the increased rate of collagen biosynthesis [118]. The abnormal expression of the PLOD family promotes tumor progression and metastasis in HCC patients, making PLODs potential treatment targets. Meanwhile, scientific studies have shown that PLODs’ expression correlates with immune cell infiltration. Overall, PLODs are responsible for balancing the hepatic microenvironment, especially in HCC [116]. In a recent study, LH3 was found to recruit matrix metalloproteinase 9, which is involved in ECM remodeling and TGF-β activation [117]. Banushi et al. has shown that VIPAR, a trafficking protein, is responsible for regulating the interaction of LH3 and collagen-containing organelles from the trans-Golgi network (TGN). VIPAR deficiency causes arthrogryposis, renal dysfunction, and cholestasis syndrome (ARC), which involves functional defects throughout several organs, including the liver. A surprising feature of this syndrome is that some clinical phenotypes overlap with those found in patients with inherited LH3 deficiencies [117].

- Catalase (CAT)

The CAT gene encodes catalase, an essential enzyme located in the peroxisomes of almost all aerobic cells, which breakdowns the intracellular H_2_O_2_ into water and oxygen without generating free radicals. Studies have shown that oxidative stress contributes to the pathogenesis of cholestasis via cytokines, and it is believed that tissue injury in cholestatic patients is caused by lipid peroxidation [91,119]. A complex antioxidative system such as catalase is activated in the presence of free oxygen radicals, so it makes sense that mean catalase levels are reported to be significantly higher in hepatocytes of cholestatic patients than in controls. Catalase is involved in defining hepatic antioxidant capacity and is increased in many hepatic injuries, especially cholestasis, which is due to increased demand for hepatocyte detoxification from excess production of H_2_O_2_ [120]. There are approved mechanisms for the flavonoid-rich extract of *Hypericum* against H_2_O_2_-induced apoptosis in PC12 cells, including decreased H_2_O_2_ cytotoxicity, and reduced DNA laddering (Figure 5D) [121].

- Bcl-2-Associated X-protein (BAX)

The BAX gene encodes a 21 kDa pro-apoptotic protein, named Bcl-2-Associated X-protein (BAX), an important member of the Bcl-2 gene family, which regulates intrinsic apoptosis [122]. The intrinsic pathway of apoptosis is triggered by free radicals, toxins, and impaired DNA [123]. Cholestasis can cause hepatocyte and cholangiocyte apoptosis and necrosis. Following oxidative stress and increased accumulation of ROS, cholestasis induces BAX expression. BAX, as a pro-apoptotic factor, activates caspases and releases mitochondrial inter-membrane space (IMS). In general, cholestasis alters the expression of apoptosis-related proteins, including Bax and Bcl-2. An elevated BAX level and apoptotic body index are observed in the liver of cholestatic patients (Figure 5D) [123]. There is a proposed anti-apoptotic mechanism for hyperforin salt, including the down-regulation of BAX protein, which helps with the amelioration of solid tumors [124]. *Hypericum* ethanolic extract can regulate BAX expression, which can be considered one of the anti-cholestatic mechanisms of this precious herb. Upon increased toxin and ROS formation following cholestasis, brain damage is expected. There are studies evaluating the positive effect of neuroprotective treatments on cholestasis-induced brain injury [123]. *Hypericum*, as an anti-oxidant that exerts neuroprotective effects, may also have potential benefits on central nervous system injury second to cholestasis.

- Chemokine (C-X-C motif) ligand 2 (CXCL2)

Bile acids induce CXCL2 expression, which recruits neutrophils to inflamed sites during cholestasis [99,125]. CXCL2 is a secreted chemokine that belongs to the chemokine superfamily and plays an important role in immunoregulation and inflammatory processes [126]. The TLR2/S100A8/S100A9 signaling pathway is introduced as a hepatic CXCL2 regulator, which results in neutrophil recruitment (Figure 5E) [127]. Neutrophil production is regulated via granulocyte-colony-stimulating factor (G-CSF), and CXCL2 as a chemokine. In response to CXCL2 gradients and other stimuli, mature neutrophils migrate into inflamed sites [104]. Several studies have reported increased levels of CXCL2 and its receptor (CXCR2) after bile duct ligation (BDL) and subsequent liver injury. The activation of this pathway leads to the formation of chemokine gradients, neutrophil recruitment, and regulation of hepatocyte proliferation and death during liver damage [128]. There is a biological link between hepatic c-Jun NH2-terminal kinase (JNK), the AP1 signal transduction pathway, and CXCL2 [129]. This axis is regarded as being responsible for insulin resistance and hepatic steatosis. The JNK axis inhibits PPARα which is an important lipid and bile acid hemostasis regulator, affecting intracellular fatty acid transport. The JNK axis blockade activates PPARα in hepatocytes which modulate bile acid hemostasis. Several studies have introduced this axis as a therapeutic target for treating cholestasis and hepatic steatosis [130]. Studies have shown the beneficial effects of low-dose fenofibrate on the JNK-AP1-CXCL2 axis and subsequent PPARα activation, which ultimately ameliorates cholestatic liver [131]. Bile acid regulates bile metabolism by activating the JNK signaling pathway and therefore downregulating CYP7A1, which ultimately leads to bile acid over-production and cholestasis [131].

- Enhancer of zeste homolog 2 (EZH2)

EZH2, a histone methyltransferase (HMT) subunit of the polycomb repressive complex 2 (PRC2), is a key regulator of downstream gene expression. EZH2 has an essential role in silencing tumor-suppressive microRNAs and its overexpression is correlated with the development of solid tumors such as HCC. EZH2 overexpression inhibits insulin-like growth factor binding protein 4 (IGFBP4), which is normally expressed in the liver and functions as a tumor suppressor. In addition to being a diagnostic biomarker of HCC, EZH2 is also considered a good therapeutic target for this disease. EZH2 inhibitors reduce serum levels of inflammatory cytokines, including IL-6, IL-1β, and IFN-γ [132,133]. EZH2 regulates cellular cholesterol homeostasis via DNA methyltransferase 1 (DNMT1) recruitment and downregulation of ATP-binding cassette transporter A1 (ABCA1). ABCA1 exerts atheroprotection properties and is mainly expressed in macrophages and hepatocytes. EZH2-induced DNMT1 recruitment in macrophage-derived foam cells promotes CpG methylation of the ABCA1 gene, which ultimately impairs cholesterol efflux and leads to atherosclerosis [134].

- Sterol Carrier Protein 2 (SCP2)

**Figure 6:**
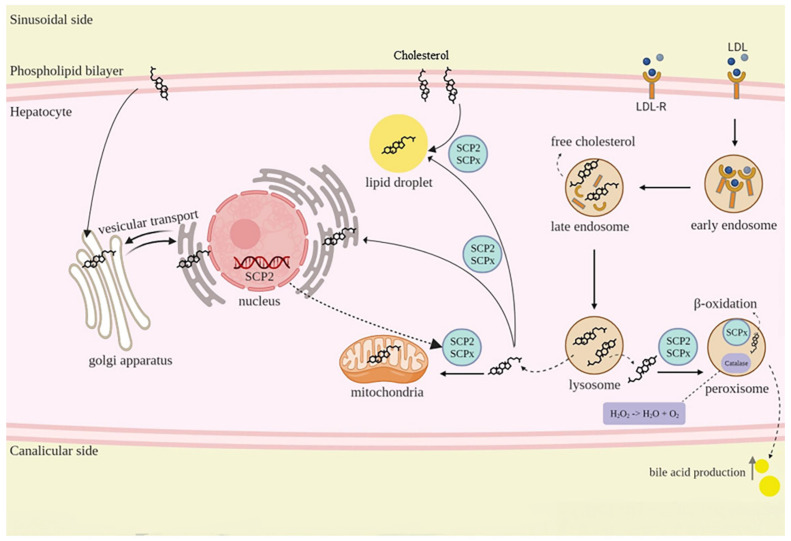
SCP2/SCPx function in cholestasis. LDL transport from the sinusoidal side to the hepatocyte via the LDL receptor (LDL-R). Due to the acidic pH of endosomes, LDL-R breaks into free cholesterol. The transportation of free cholesterol between different organelles, including mitochondria, plasma membrane, peroxisome, and lysosomes is facilitated by SCP2/SCPx. The entrance of free cholesterol into the lipid droplet from the sinusoidal side is also carried out via SCP2/SCPx. Apart from these, SCPx is a key factor in peroxisomal β-oxidation of fatty acids and bile acid production.

SCP2 encodes two distinct isoforms, SCP2 and SCPx, which have a common 13 kDa carboxy terminus. It has been found that the amino acid sequence of SCP2/SCPx is conserved across different species, indicating that these two proteins serve important functions. The 58 kDa protein (SCPx) is localized to peroxisomes [135,136] and serves a catalytic role as a thiolase in cholesterol biosynthesis. Studies have shown that SCPx expression is sex-dependent; male murine models of puberty show an increase in hepatic levels of SCPx, while female murine models show a decrease. The fact that SCPx expression is specifically controlled by androgens during sexual maturation suggests that androgens play a significant role in controlling SCPx expression [137]. In diseases associated with impaired bile acid synthesis and cholesterol binding, the gene is highly expressed [138]. The liver plays a key role in regulating the cholesterol synthesis rate. Furthermore, it coordinates the removal of cholesterol from the body by either removing it directly into the bile or converting it into more water-soluble bile acids [139]. Free cholesterol (FC) or non-esterified cholesterol cannot move between cell membranes or within cellular compartments due to its hydrophobicity. Intracellular cholesterol transport is facilitated by intracellular lipid transporter proteins such as SCP2 (13 kDa) (Figure 6). The expression of this carrier is induced by *Hypericum* and is closely correlated with the rate of intracellular cholesterol metabolism in different tissues, especially the liver, which maintains cholesterol homeostasis. He et al. evaluated the effect of SCP2/SCPx deficiency on intestinal cholesterol absorption [138]. There is a close correlation between decreased levels of hepatic lipid transport proteins, including SCPx and SCP2 and Niemann–Pick type C (NPC) disease, which is characterized by accumulated hepatic cholesterol in lysosomes and Golgi [138]. As lysosomal cholesterol is the primary precursor of androgens, a deficiency of intracellular cholesterol transport from lysosomes in NPC patients is responsible for disrupting androgen production [140].

- Cyclin-dependent kinase 6 (CDK6)

CDK6 is a member of the CDK family that is generally responsible for regulating DNA replication. CDKs are characterized as essential signaling factors for hepatocyte proliferation in the late gestation period of fetal liver development [141]. CDK6 is a protein-coding gene that encodes a 37 kDa serine/threonine protein kinase. The encoded kinase is a mitogenic factor, in complex with D-type cyclins (D1, D2, and D3 cyclins), promoting G1 phase progression and G1/S transition, and is ubiquitously expressed. The CDK6-cyclin complex regulates retinoblastoma (Rb), a tumor suppressor protein, and therefore plays a key role in the early phases of several human cancers. Rb will release E2F, a transcription activator of necessary genes for DNA replication when the CDK-cyclin complex phosphorylates it. The Rb-E2F pathway exerts a key role in the regulation of cell proliferation and differentiation during the G1 phase of the cell cycle. Overexpression of CDK6 will lead to phosphorylation and activation of the Rb-E2F regulating pathway. Rb phosphorylation can be controlled by members of the INK family and the CIP/KIP family [142]. It has been observed that bile acids may cause DNA damage to hepatocytes in previous studies [143]. As a result, the upregulation of CDK6 by *H. perforatum* can be considered a hepatoprotective mechanism against cholestasis.

- Superkiller Viralicidic Activity 2-Like (SKIV2L)

The human super killer (SKI) complex works alongside RNA exosomes to mediate RNA surveillance. The human SKI complex is made up of four proteins: TPR encoded by tetratricopeptide repeat domain-containing protein 37 (TTC37), two WD40-containing subunits encoded by WDR61, and SKI2W which is encoded by super killer viralicidic activity 2 (SKIV2L) [144]. It is believed that pathogenic variants in SKIV2L and TTC37 often relate to a rare recessive genetic disorder known as trichohepatoenteric syndrome (THES). A third of THES cases are reported to be caused by SKIV2L gene mutations, and another third are caused by TTC37 gene mutations [145]. In a recent study, severe infantile liver disease has been associated with a novel homozygous frame-shift mutation (c.3391 delC) in SKIV2L [146]. The syndrome is characterized by intractable diarrhea, hepatic problems, facial and hair dysmorphisms, and intellectual disabilities in the first few months of life. These patients often suffer from cardiac abnormalities and immune dysregulations. Patients with SKIV2L mutations may benefit from growth hormone therapy [145]. Therefore, SKIV2L expression under *Hypericum* treatment could be involved in regulating bile flow and hepatic hemostasis.

- NISCH

The NISCH encodes imidazoline receptor antisera-selected (IRAS), also known as nischarin, which is a novel protein interacting with α5 cytoplasmic domain of integrins, and therefore affects cell migration and ECM microarchitecture [147]. It was claimed that nischarin may function as an imidazoline 1 receptor (I1R) in some cells [148]. However, this hypothesis was later challenged by Arnoux et al., who concluded that NISCH and I1R are not the same [149]. Liver fibrosis, as a life-threatening result of cholestasis, is progressed by releasing of pro-inflammatory cytokines, activation of HSCs, and Kupffer cells. Activation of I1R serves a hepatoprotective role in the fibrotic liver by interacting with the Nrf2 signaling pathway. Activation of the Nrf2 signaling pathway is associated with the inhibition of the TLR4/NF-*k*B pathway and TGF-β, as one of the most important cytokines related to fibrosis [150].

- Conserved oligomeric Golgi (COG 4,7)

COG encodes conserved oligomeric Golgi complex with eight subunits, and exerts its role in the maintenance of Golgi apparatus structure and function. COG4 is one of the subunits and is located in lobe A of this complex and COG7 is located in lobe B [151]. The Golgi apparatus is an important site for protein and lipid glycosylation [152]. There is a reported vesicular pathway of transportation of bile acid into the hepatocyte which mainly involves the Golgi apparatus. After the uptake of bile acids by hepatocyte sinusoidal plasma membrane, they bind to proteins. The complex formed (bile acid + protein) can be taken up by the Golgi apparatus and move towards the canalicular membrane via Golgi-derived vesicles [153]. It can be hypothesized that any defect in genes that are related to the Golgi apparatus maintenance and function, including COGs, can lead to impairment in bile flow and ultimately cholestasis.

### 2.5. Interacting Network of Hypericum Metabolites and Their Target Genes

**Figure 7:**
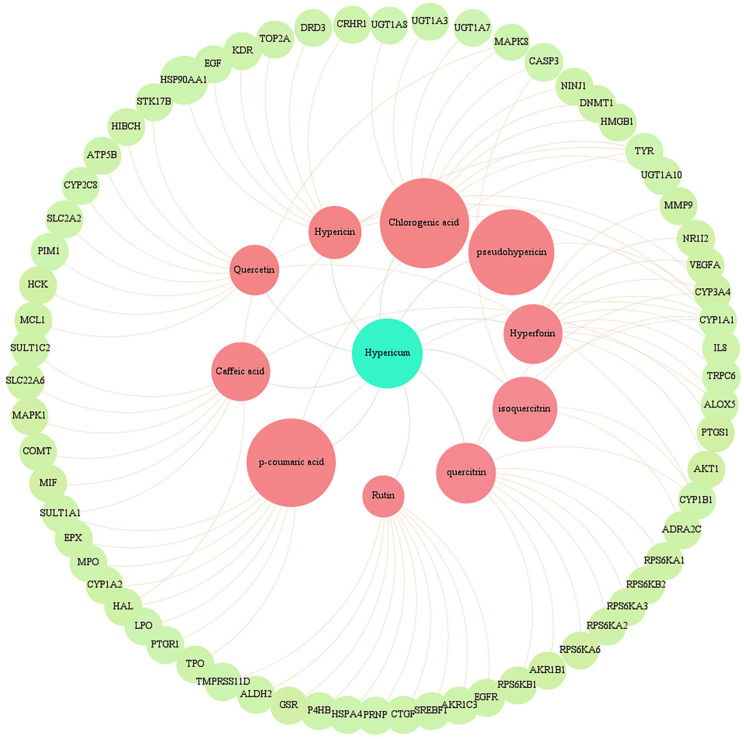
Potential target genes against cholestasis associated with each of the *Hypericum perforatum* L. bioactive metabolites. Hypericin, hyperforin, quercitrin, isoquercitrin, caffeic acid, *p*-coumaric acid, chlorogenic acid, rutin, quercetin, and pseudohypericin are located as central nodes.

As mentioned earlier, *Hypericum* contains several metabolites, with a broad-spectrum pharmacological attribute. In order to define the potential target genes of the named bioactive constituents, we used the STITCH database [154]. Bioactive compounds were uploaded to STITCH in order to achieve hub genes of each specialized metabolite. The organism was limited to “homo sapiens”. Only targets with an interaction score >0.4 were selected for subsequent analysis. The intersection of target genes for each *Hypericum* metabolite and cholestasis-related genes (obtained from GeneCards) were determined [155]. Retrieved target genes for each metabolite are classified in Table S3 and those that are involved in cholestasis are displayed in Figure 7. A full description of the genes that are mainly involved in hepatobiliary disorders is discussed below.

Caffeic acid is capable of affecting MAPK1/8 expression, which is involved in liver toxicity during cholestasis [92]. Caffeic acid, hyperforin, and hypericin have the capacity to affect arachidonate 5-lipoxygenase (ALOX5) expression, which is believed to accelerate atherosclerosis by elevated leukotriene synthesis via leukocytes and therefore increased inflammation within arterial walls [156]. As observed in the interaction network (Figure 7), caffeic acid can also regulate the expression of organic anion transporter 1 (OAT1/SLC22A6) involved in the regulation of bile acid secretion and transportation. This is in agreement with experimental observations that demonstrate a decreased serum level of bile acid compared to the wild-type in OAT1 knockout mice [157].

Chlorogenic acid also alters MAPK8 regulation, according to the interacting network of *Hypericum* metabolites and target genes. Chlorogenic acid and kaempferol are capable of changing UGT1A3/7/8/10 expression, which encodes the enzyme UDP-glucuronosyltransferase (UGT). Any defect in UGT expression, as the bile acid detoxifier, is believed to be related to the accumulation of toxic molecules in the body, including bile acids [158]. Chlorogenic acid also regulates DNMT1 expression, which is involved in the impairment of cholesterol efflux as mentioned earlier. This metabolite regulates high-mobility group box 1 (HMGB1) expression, which is released after liver injury, activates HSCs, and ultimately induces liver fibrosis as a repair response [159].

As observed in Figure 7, the expression of CYP1A1 is inhibited by several metabolites, including hypericin, hyperforin, pseudohypericin, quercitrin, quercetin, and *p*-coumaric acid. This observation is in agreement with previous studies conducted on *Hypericum* extract [160]. Previous studies have shown that CYP1A1 is upregulated by bile acids, which may explain the carcinogenesis effect of cholestasis [161]. Generally, CYP1A (including CYP1A1/2) is found to regulate cholesterol biosynthesis pathways which provide new insights into the treatment of hypercholesterolemia. *p*-coumaric acid, a main metabolite of *Hypericum*, affects CYP1A2 expression and is suitable to regulate cholesterol homeostasis [162]. The expression of myeloperoxidase (MPO), an enzyme related to oxidative stress, inflammation, and liver fibrosis, is affected by *p*-coumaric acid [163]. Elevated MPO activity in hepatic neutrophils has shown correlations with hepatocyte damage and nonalcoholic steatohepatitis (NASH) [164]. 

The interacting network of metabolites and target genes shows that hypericin, hyperforin, and quercitrin affect CYP3A4 expression (Figure 7), which is in agreement with the previous studies stating that *H. perforatum* induces CYP3A4 [165]. CYP3A4, as the potential bile acid detoxifier, is recommended as one of the therapeutic mechanisms against cholestasis facilitating the consequent bile acid glucuronidation reaction by UGTs [166]. Other CYPs, such as CYP1B1 and CYP2C8, can be potential targets for the treatment of metabolic diseases [167]. CYP1B1 is targeted by quercitrin, isoquercitrin, rutin, quercetin, and kaempferol. The interaction network shows that quercetin, a common flavonol aglycone, affects CYP2C8 and CYP1B1 expression. In addition, quercetin affects anti-apoptotic Mcl-1, which exerts hepatoprotective effects following cholestasis-induced bile duct ligation. Overexpressing hepatic Mcl-1 is a suggested therapeutic mechanism against liver injury [168].

Vascular endothelial growth factor A (VEGFA) from hepatocytes is contributed to NAFLD with the mechanism of activating HSCs in the liver, so it can be counted as a good therapeutic target against NAFLD [169]. Antiangiogenic therapies have been evaluated as a good therapeutic choice for improving chronic liver disease-induced cholestasis [170]. Interestingly, hyperforin shows also antiangiogenic activity by inhibiting VEGFA [171]. According to the identified hub genes, both hyperforin and hypericin target VEGFA (Figure 7, Table S3). Matrix metalloproteinase 9 (MMP9), involved in hepatic tissue repair, remodeling, and cellular movement, is regulated by hyperforin, hypericin, quercetin, and luteolin. MMP9 can be secreted from Kupffer cells and HSCs, and induce the release of growth factors from ECM [172].

There is evidence that heat shock protein 90 (HSP90) is associated with liver injury, and HSP90 inhibitors serve as protectors of several organs. Administration of 17-DMAG, an HSP90 inhibitor, was related to the amelioration of cholestasis and the reduction of IL-1β and IL-18 expression [173]. Amongst *Hypericum* metabolites, hypericin affects HSP90 expression, which is worth further in-depth investigation (Figure 7). 

## 3. Materials and Methods

In this article, the key points of pharmacological mechanisms of *Hypericum* choleretic and anti-cholestasis characteristics appear in two distinct sections as follows:

### 3.1. Hypericum Bioactive Components and Their Pharmacological Attributes with an Emphasis on Cholestasis Found in Literature

First, we searched medical attributes to *H. perforatum* known as “Houfariqun” with a phonetic spelling as/hu:fɑ:rɪɢu:n/in Makhzan-ol-advieh, a traditional Persian pharmacopeia collected by “Aqili al-Alavi” (1772 A.D.) to investigate its traditional medicinal properties. Then, different terms, including “*Hypericum perforatum*”, “St. John’s wort”, “Cholestasis”, “bile acid”, “pharmacological mechanisms”, and “bioactive components”, were searched through engines such as “PubMed” and “Google Scholar” until September 2022.

### 3.2. Potential H. perforatum Target Genes against Cholestasis

*H. perforatum* expression microarray data sets were extracted using the GEO website (http://www.ncbi.nlm.nih.gov/geo/, accessed on 1 May 2023). The study type was restricted to expression profiling by an array. The GEO website contains three expression microarray datasets for *H. perforatum* L., including GSE56571, GSE21841, and GSE144235 [174,175,176]. Since sample numbers were limited for GSE56571 and GSE21841, we only selected GSE144235 [174]. To investigate the potential target genes related to cholestasis, we used GSE144235 with 228 samples containing 48 controls [174]. The transcriptional profile of GSE144235 was retrieved via Genometry L1000™ Expression Profiling using four human cell lines, including MCF7, Ishikawa, HepG2, and A549, to evaluate the toxicity of a dietary supplement containing *Hypericum*, ginger, kava kava, chaste tree, and ashwagandha [174]. We have performed an in-depth analysis on HepG2 cells that were treated with three different concentrations of *H. perforatum* extract (2.5, 25, 250 µg/mL) or DMSO as a vehicle for a 6-h time point. Notably, *Hypericum* extracts with a concentration of 25 and 250 µg/ml were defined to be cytotoxic according to VanderMolen et al. Therefore, we have intensively considered hepatocytes treated with 2.5 µg/ml *Hypericum* extract. The differentially expressed genes (DEGs) were identified and analyzed using GEO2R. Benjamini–Hochberg false discovery rate was used to adjust the *p*-value. |log Fc (fold change)| > 0.2 and a *p*-value < 0.05 were set as statistical significance criteria. 

To acquire genes involved in cholestasis, we used “cholestasis” as the keyword to search in GeneCards as a human gene database [177]. Genes with “gifts > 30” were selected as potential genes involved in cholestasis. The intersections of DEGs upon treatment with *Hypericum* extract and potential genes that are involved in cholestasis were generated and defined using the Bioinformatics & Evolutionary Genomics online tool (https://bioinformatics.psb.ugent.be/webtools/Venn/), accessed on 1 May 2023, to display the Venn diagram. Overlapped DEGs were used to define hub genes. The DAVID online analysis tool, which is a database for annotation, visualization, and integrated discovery was used for gene ontology (GO) enrichment analysis [178].

To retrieve a protein–protein interaction (PPI) network by defining Homo sapiens as the target organism, we used “String”, a search tool for the retrieval of interacting genes and proteins [179]. The protein interaction network of DEGs was constructed by Cytoscape software [180]. The Cytohubba plug-in was utilized in order to introduce potential hub genes [181]. Then, each gene was searched using different terms, including “cholestasis”, “cholagogue”, “bile acid”, and “cholesterol”. Finally, in order to predict potential target genes associated with each *Hypericum* metabolite, we used the STITCH database [154]. The main secondary metabolites of *Hypericum* as bioactive compounds were uploaded to STITCH using the SMILE format in order to achieve the target genes of each bioactive component. The organism was limited to “Homo sapiens”. Only targets with an interaction score >0.4 were selected for subsequent analysis. The cholestasis-related genes obtained from GeneCards and the predicted genes of each *Hypericum* bioactive metabolite were intersected to define the target gene for each metabolite [155].

## 4. Conclusions

Effective therapeutics against hepatic disorders are worryingly limited and might eventually result in liver transplantation with a high mortality rate. Therefore, hepatic afflictions can be considered complex disorders which require multi-targeted drug discovery. One of the main therapeutic approaches in treating cholestasis is increasing the bile flow (choleretic effect) and excreting excess bile by feces (cholagogue effect). *Hypericum* helps with hepatitis, hepatocyte peroxidation, NAFLD, hepatic lipid metabolism, and hepatic steatosis. *Hypericum*’s hepatoprotective activity can be attributed to the suppression of TNF-α and IL-6 cytokine contents in the liver. *Hypericum* regulates LysoPC content as a hepatic inflammation biomarker and regulates the secretion and expression of adiponectin, which balances glucose and fatty acid metabolism. *Hypericum* extract lowers total cholesterol, raises HDL, and decreases triglyceride content. As degeneration and lytic necrosis are features of cholestatic hepatitis, *Hypericum* triggers the regulatory mechanism for liver regeneration. A total of 16 common genes, including EZH2, SCP2, ILK, CDK6, EGF, SPP1, PLOD3, SKIV2L, CXCL2, NISCH, ICAM1, SRC, CAT, COG7, BAX, and COG4, which are also involved in cholestasis, were identified under the treatment with the low dose of *Hypericum* extract. *Hypericum* targets the MAPK signaling pathway, which in turn regulates various hepatic functions, including bile acid synthesis, bile acid excretion, bile-acid-induced apoptosis, and hepatic steatosis. Target genes that are affected by *Hypericum* extract or its metabolites regulate cholesterol homeostasis to control hypercholesterolemia, detoxify bile acids, regulate hepatocyte differentiation, growth, migration, and survival, and increase the wound healing ability of HSCs toward liver regeneration through the modulation of collagen production. For example, hyperforin, one of the most important specialized metabolites of *Hypericum*, shows antiangiogenic activity by inhibiting VEGFA, which is suitable for controlling NAFLD. Quercetin, which affects the anti-apoptotic Mcl-1 protein, prevents liver injury following cholestasis. Taken together, merging the data retrieved through traditional medicine with the target genes that are affected by *Hypericum* improves the treatment approaches against cholestasis and related hepatic injuries such as hepatic carcinoma. Finally, standard trials using *Hypericum* alone and in combination with UDCA as adjuvant or second-line therapy should be designed to define the placement of this plant species in the treatment of cholestasis and its symptoms.

## Figures and Tables

**Table 2:** Gene ontology and KEGG enrichment results of differentially expressed genes upon treatment with *H. perforatum* extract overlapped with cholestasis-involved genes. MF, BP, and CC, refer to molecular function, biological process, and cellular components, respectively.

Category	Term	*p*-Value	Target Gene
MF	Integrin binding	6 × 10^−7^	NISCH, SRC, SPP1, ILK, ICAM1
Bp	GO:0043406~positive regulation of MAP kinase activity	4 × 10^−4^	SRC, EGF, ILK, EZH2
	GO:0033993~response to lipid	7 × 10^−4^	SRC, CAT, SPP1, CXCL2, EZH2, ICAM1
	GO:0009893~positive regulation of metabolic process	4 × 10^−3^	CDK6, SCP2, SRC, EGF, SPP1, BAX, ILK, EZH2, ICAM1
	GO:0014065~phosphatidylinositol 3-kinase signaling	4 × 10^−3^	SRC, EGF, CAT
CC	Endomembrane system	2 × 10^−3^	NISCH, COG7, SCP2, SRC, EGF, COG4, CAT, SPP1, BAX, PLOD3
KEGG pathway	Lipid and atherosclerosis	3 × 10^−3^	SRC, BAX, CXCL2, ICAM1
	EGFR tyrosine kinase inhibitor resistance	5 × 10^−3^	SRC, BAX, EGF

## Data Availability

Data sharing not applicable.

## Supplementary Materials

The following supporting information can be downloaded at: https://www.mdpi.com/article/10.3390/ph16060887/s1: Table S1: Differentially expressed genes in HepG2 cells under the treatment with 2.5 µg/ml *Hypericum perforatum* extract; Table S2: The involved genes in cholestasis; Table S3: Predicted target genes affected by *Hypericum perforatum* metabolites.
